# Comprehensive safety assessment of donepezil: pharmacovigilance analysis based on the FDA adverse event reporting system

**DOI:** 10.3389/fneur.2025.1655216

**Published:** 2025-12-10

**Authors:** Shuo Li, Wenlong Qian, Zhuo Zhang, Yonghua Chen, Xiaoxiao Hou, Bingjie Min, Hui Zhou, Xinyu Zhu, Jia Ling, Wenming Yang, Shijian Cao

**Affiliations:** 1 Department of Neurology, The First Affiliated Hospital of Anhui University of Chinese Medicine, Hefei, Anhui, China; 2 Center for Xin'an Medicine and Modernization of Traditional Chinese Medicine, Institute of Health and Medicine Hefei Comprehensive National Science Center, Hefei, Anhui, China; 3 Key Laboratory of Xin'An Medicine, Ministry of Education, Anhui, China

**Keywords:** donepezil, FDA adverse event reporting system, disproportionality analyses, pharmacovigilance, adverse event

## Abstract

**Background:**

Alzheimer’s disease (AD) has a growing global prevalence, and the need for safe and effective treatments is urgent. Donepezil is commonly used therapeutic agents for AD but has safety controversies. The objective of this study was to thoroughly evaluate donepezil’s adverse event profile using actual data.

**Methods:**

In this study, reports of donepezil-related adverse events were collected from the first quarter of 2004 to the fourth quarter of 2024 through the FAERS database. The association of donepezil-induced adverse events was disproportionality analyzed using Reporting odds Ratios (ROR) and Proportional Reporting Ratio (PRR) and Bayesian Confidence Propagation Neural Network (BCPNN) and Multi-item Gamma Poisson Shrinker (MGPS), among other methods.

**Results:**

A total of 26,120 ADRs with donepezil as the “first suspect” were retrieved during the reporting period. The most common AEs included nausea, vomiting, syncope, and dizziness, which were consistent with the labeling of the medication and clinical trials. Unintended major AEs such as fall, hypotension, tremor, cognitive disorder, mania, and the highest signal of pleurothotonus were also detected. The reports also collected were characterized by a high proportion of female patients (51.3%) and the time of AE induction within 30 days (41%).

**Conclusion:**

Donepezil treatment needs to focus on cardiovascular and neurological adverse events, especially for women, elderly patients, or patients with co-morbidities, cardiac monitoring and dose adjustment should be strengthened. Clinics need to balance efficacy and risk, develop individualized dosing regimens, and explore novel therapeutic strategies to improve long-term safety.

## 1 Introduction

Alzheimer’s disease (AD) is recognized as the most common form of dementia and is widely known to be a prevalent neurodegenerative disorder. It is classified as a degenerative lesion that affects the central nervous system and is marked by the progressive worsening of cognitive functions and significant behavioral impairments. The accumulation of beta-amyloid peptide (Aβ) in the brain leads to the formation of neuroinflammatory plaques and tangles of neuroprogenitor fibers. These abnormalities primarily affect critical brain regions, including the medial temporal lobe and neocortical structures, therefore contributing to the defining characteristics of the disease (1). The clinical presentation is characterized by a wide range of symptoms, including memory impairment, aphasia, dysarthria, dyscognition, deficits in visuospatial abilities, difficulties with abstract thinking and calculation, and significant personality and behavioral changes (2, 3). The pathogenesis of AD is acknowledged to be complex and multifactorial, involving various mechanisms such as selective autophagy dysfunction and mitochondrial impairment. The pathogenic processes are influenced by the activation of the NLRP3 inflammasome and alterations in insulin signaling, including insulin resistance. Selective autophagy is believed to be an active pathogenic factor in AD progression. Therefore, targeting and modulating this specific autophagic pathway may represent a promising therapeutic strategy for intervening in the course of AD (4).

As the global population continues to age at an accelerated pace, more individuals are developing Alzheimer’s disease each year. The International Alzheimer’s Disease Association has reported that, in 2018, around 50 million people across the world were living with AD. Projections suggest that this number could triple by 2050, underscoring the urgent need for increased awareness, research, and intervention strategies (5). Moreover, the population affected by AD predominantly comprises older adults, making aging a prominent feature of the condition. Advanced age is widely regarded as one of the most critical risk factors for the onset of AD. This strong association with age contributes to the disease’s high mortality rate, with AD currently listed as the sixth leading cause of death in the general population and the fifth among individuals over 65 years old (6).

The clinical management of AD is currently based on four primary classes of pharmacological agents: donepezil, rivastigmine, galantamine, and memantine. These medications work by modulating the function of the cholinergic and glutamatergic neurotransmitter systems. Treatment regimens may involve either single-drug therapy or a combination of agents (7). Acetylcholine (ACh) synthesis reduction is considered the most critical biochemical change underlying AD. This deficit, along with the degeneration of cholinergic neurons and the loss of effective neurotransmission in the brain, has been identified as the principal mechanism leading to the cognitive impairments characteristic of the disease (8). The Acetylcholinesterase (AChE) terminates synaptic signaling by breaking down ACh through hydrolysis. The AChE-S tetramer isoform plays a central role in regulating cholinergic activity in the brain, as it anchors to neuronal membrane proteins and influences synaptic transmission. The cholinesterase inhibitors (ChEIs) help improve cognition in AD by actively and reversibly blocking AChE. This inhibition increases the time ACh remains active in the synaptic gap, thus enhancing cholinergic signaling (9). Acetylcholinesterase inhibitors (AChEIs) have also emerged as first-line agents for AD treatment.

Donepezil is recognized as a commonly used acetylcholinesterase inhibitor in the clinical management of AD. Although its efficacy in improving cognitive function and delaying disease progression in AD patients has been well established, its clinical application remains associated with several AEs that warrant attention. Common AEs, including nausea, diarrhea, insomnia, muscle spasms, fatigue, anorexia, headache, and vomiting, have been reported in clinical phase III trials and long-term observational studies of donepezil. Moreover, the FDA specifically labels donepezil with a warning regarding the serious risk of bradycardia, particularly when combined with beta-blockers or other heart conduction drugs, as these combinations may cause fainting, falls, or even life-threatening atrioventricular block (10). Therefore, data mining techniques to identify potential adverse reaction signals associated with donepezil in real-world clinical settings are essential for guiding clinical decision-making.

The U.S. Food and Drug Administration Adverse Event Reporting System (FAERS) is a primary global resource for monitoring post-marketing drug safety. Integrating standardized real-world medical data plays a critical role in supporting correlation studies between drugs and AEs (11). This study, based on the FAERS database, utilizes a retrospective pharmacovigilance design and employs proportional imbalance analysis to explore potential safety signals associated with the AD treatment drug donepezil. The study aims to identify unintended AEs not yet included in the official drug label, improve its safety assessment, and provide evidence for optimizing clinical use and developing effective risk management strategies. In this study, ‘novel’ or ‘unintended’ adverse events refer to those not currently listed in the official FDA-approved prescribing information for donepezil. Identifying such signals is critical as they may reveal previously unrecognized risks, potentially influencing treatment adherence, dose adjustment, or the need for additional monitoring in clinical practice, thereby enhancing patient safety.

## 2 Materials and methods

### 2.1 Data source and processing

**Figure 1:**
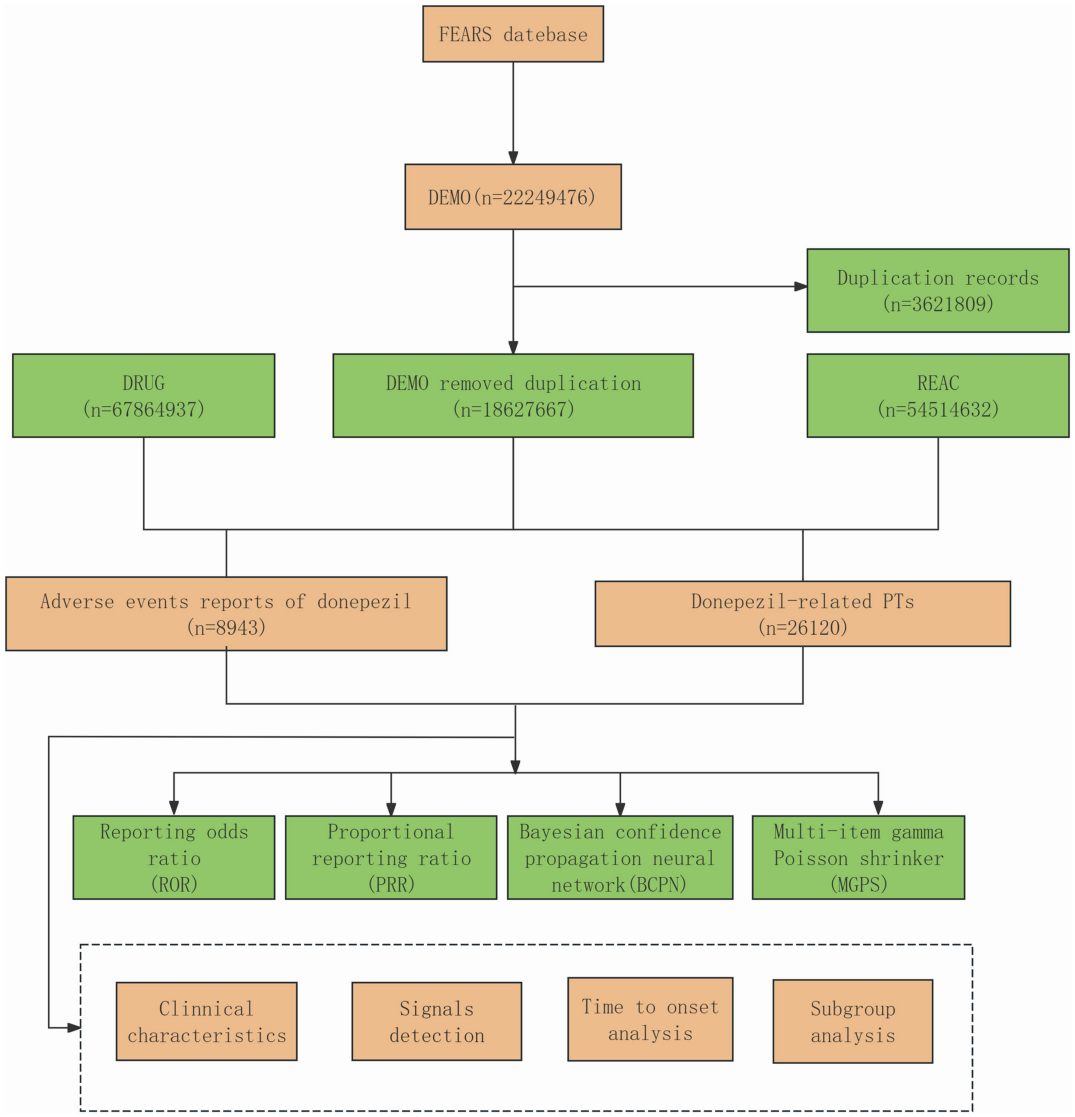
Flowchart of identifying AE cases of donepezil from the FAERS datebase.

Post-marketing adverse drug reaction surveillance in the United States is primarily conducted through FAERS, which is also regarded as one of the leading platforms for ongoing pharmacovigilance research (12). In the present drug safety monitoring investigation, ASCII-formatted case reports from Q1 2004 to Q4 2024 were extracted from the pharmacovigilance database. After extraction, the datasets were cleaned and analyzed using R version 4.4.2 and Microsoft Excel. Meanwhile we used ORIGIN2024, R software and http://113.44.3.163/ website for picture drawing. Sample groups were determined through the screening of drug names and PROD_AI entries, with “donepezil” as the designated keyword and inclusion limited to drugs classified as “primarily suspicious.” The AEs related to donepezil were coded according to the latest MedDRA 27.0 dictionary at the Preferred Term (PT) and System Organ Class (SOC) levels, and the drug’s toxicity profile was systematically evaluated. Following FDA recommendations, the PRIMARYID, CASEID, and FDA_DT fields from the DEMO table were selected to remove duplicate reports. The dataset was sorted by CASEID, FDA_DT, and PRIMARYID, and for ICSRs with identical CASEIDs, the report with the highest FDA_DT was retained. If multiple reports shared both CASEID and FDA_DT, the one with the highest PRIMARYID was selected. After eliminating duplicates, reports were further removed based on CASEIDs from the previously identified list. The data synthesis screening program is shown in Figure 1.

### 2.2 Onset time analysis

The interval between the initiation of donepezil therapy and the occurrence of AEs was calculated to determine the time to onset. Cases in which the adverse event preceded the initiation of donepezil were excluded from the dataset. The onset time was subsequently described using the median and interquartile range.

### 2.3 Statistical analysis

This study presents the reported characteristics of all donepezil-related AEs through descriptive analysis. Proportional imbalance analyses, including proportional reporting ratio (PRR) (13) and reported odds ratios (ROR) (14) and Bayesian confidence propagation neural network (BCPNN) (15) and multinomial gamma Poisson shrinkage (MGPS) (16) were utilized in our study to identify signals that indicate a possible increase in the risk of donepezil-associated adverse events. The ROR and PRR are classified as frequency methods, offering high sensitivity but low specificity. However, BCPNN and MGPS, which fall under Bayesian methods, are better suited for handling complex variables, although they have lower sensitivity (17). Therefore, this research integrates multiple computational models to ensure the robustness of the results. Higher parameter values directly correlated with stronger signal intensity, where the relevant value represents signal strength. The formulas and criteria for the four algorithms are detailed in Table 1 (18). The AEs not mentioned in the latest version of the Donepezil insert (19) published by the U.S. Food and Drug Administration (FDA) were defined as unintended adverse events.

**Table 1:** The specific formulas for the four algorithms.

Algorithms	Equation	Criteria	
ROR	ROR = ad/bc	Lower limit of 95%CI > 1, a ≥ 3	
	95%CI = e^ln(ROR) ± 1.96(1/a + 1/b + 1/c + 1/d)^0.5^		
PRR	PRR = a(c + d)/c/(a + b)	PRR > 2, X^2^ > 4, a > 3	
	X^2^ = [(ad-bc)2](a + b + c + d)/[(a + b) (c + d) (a + c) (b + d)]		
BCPNN	IC = log2a (a + b + c + d) (a + c) (a + b)	IC025 > 0	
	95% CI = e^ln(IC) ± 1.96(1/a + 1/b + 1/c + 1/d)^0.5^		
MGPS	EBGM = a (a + b + c + d)/(a + c)/(a + b)	EBGM05 > 2, N > 0	
	95%CI = e^ln(EBGM) ± 1.96(1/a + 1/b + 1/c + 1/d)^0.5^		
The fourfold table of disproportionality measures	Target AE	OtherAE	Total
Target drugs	a	b	a + b
Other drugs	c	d	c + d
Total	a + c	b + d	a + b + c + d

Equation parameters: a, reports with both the target drug and target adverse event; b, reports listing other adverse events for the target drug; c, reports documenting the target adverse event with alternative medications; d, reports involving non-target drugs and non-target adverse events. The MGPS methodology utilizes an empirical Bayesian framework, deriving prior distributions through maximum likelihood estimation, and then integrating prior and likelihood to establish posterior distributions. EBGM05 signifies the fifth percentile of this posterior distribution, representing the one-sided 95% confidence lower bound for the EBGM. 95% CI, 95% confidence interval; N, report count; χ^2^, chi-squared; IC, information component; IC025, IC’s 95% CI lower limit; E(IC), IC expectation; V(IC), IC variance; EBGM, empirical Bayesian geometric mean; EBGM05, posterior distribution’s EBGM lower confidence bound.

## 3 Results

### 3.1 Descriptive analysis

**Figure 2:**
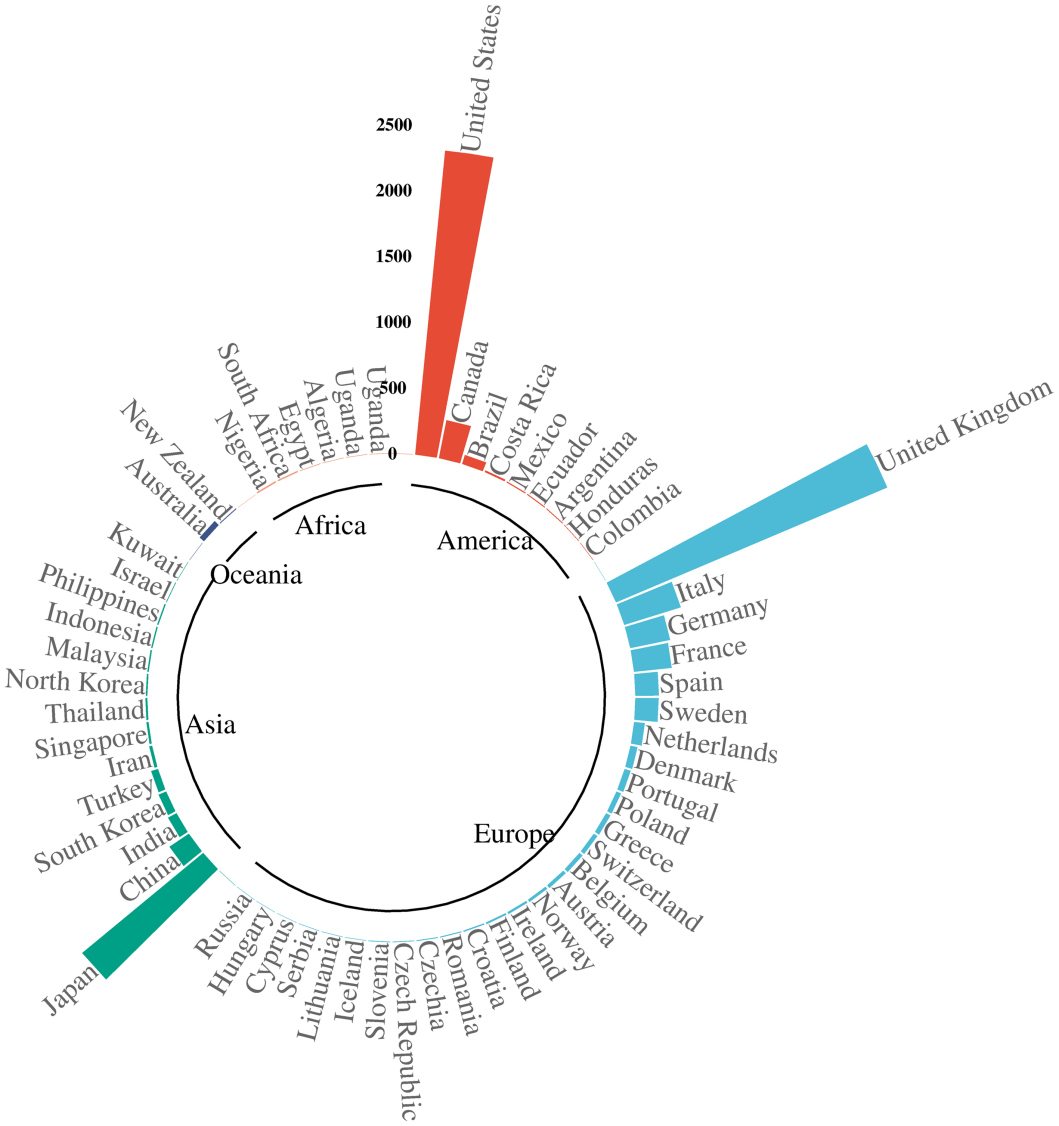
Polar Bar Chart of reporting countries.

**Figure 3:**
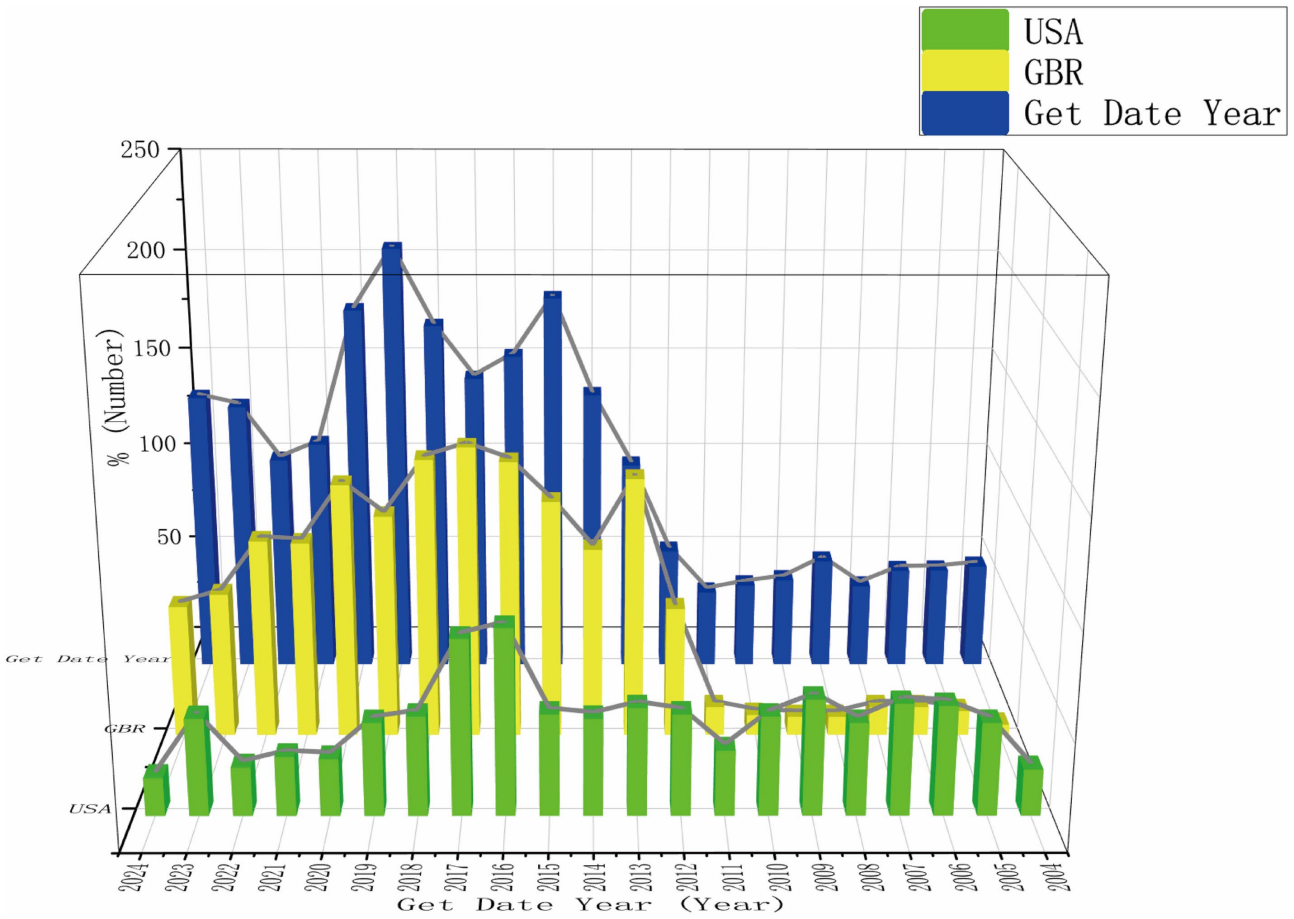
Comparison of trends in the number of reported years in high-income countries.

A total of 22,249,476 ICSRs were captured from the FAERS database during the study period, including 8,943 related to donepezil. The clinical features of these donepezil-associated events are summarized in Table 2. Among the ICSRs, more reports involved females (51.3%) compared to males (3,187). Patients aged between 65 and 85 years represented the majority, with 4,527 cases (50.6%). Most reports were submitted by pharmacists (31.7%). The peak reporting years included 2019 (848 reports), 2015 (752), and 2020 (728). Reports were largely from developed countries such as the United States (2,320; 27.4%) and the United Kingdom (2,238; 26.4%), as shown in Figure 2. After filtering the reported year data, the USA and GBR reported year data (which made up over 50%) were chosen for trend comparison. At the same time, the total reported year data by 1/4 to make the comparison data easier was reduced to understand, as seen in Figure 3.

**Table 2:** Clinical characterization of donepezil from the FAERS database.

Characteristics	ICSRs number, *n*	ICSRs proportion, %
Gender (*n* = 8,943)		
Female	4,591	51.3
Male	3,187	35.6
Unknown	1,165	13.0
Age (*n* = 8,943)		
<18	66	0.7
18–64	611	6.8
65–84	4,527	50.6
≥85	1,530	17.1
Unknown	2,209	24.7
Serious outcome (AEs case number, *n* = 8,943)		
Death	753	8.4
Hospitalization-initial or prolonged	3,462	38.7
Disability	341	3.8
Life-threatening	587	6.6
Other serious	2,460	27.5
Reported countries (Top five, *n* = 8,478)		
United States	2,320	27.4
United Kingdom	2,238	26.4
Japan	1,192	14.1
Italy	449	5.3
Germany	308	3.6
Indication (Top five, *n* = 8,943)		
Dementia Alzheimer’s type	3,025	33.8
Product Used for Unknown Indication	2,076	23.2
Dementia	1,505	16.8
Missing	736	8.2
Cognitive disorder	294	3.3
Reported person (*n*=)		
Pharmacist	1,100	31.7
Other	854	24.6
Physician	594	17.1
Healthcare professional	413	11.9
Missing	127	3.6

### 3.2 Potential safety signal detection results

**Figure 4:**
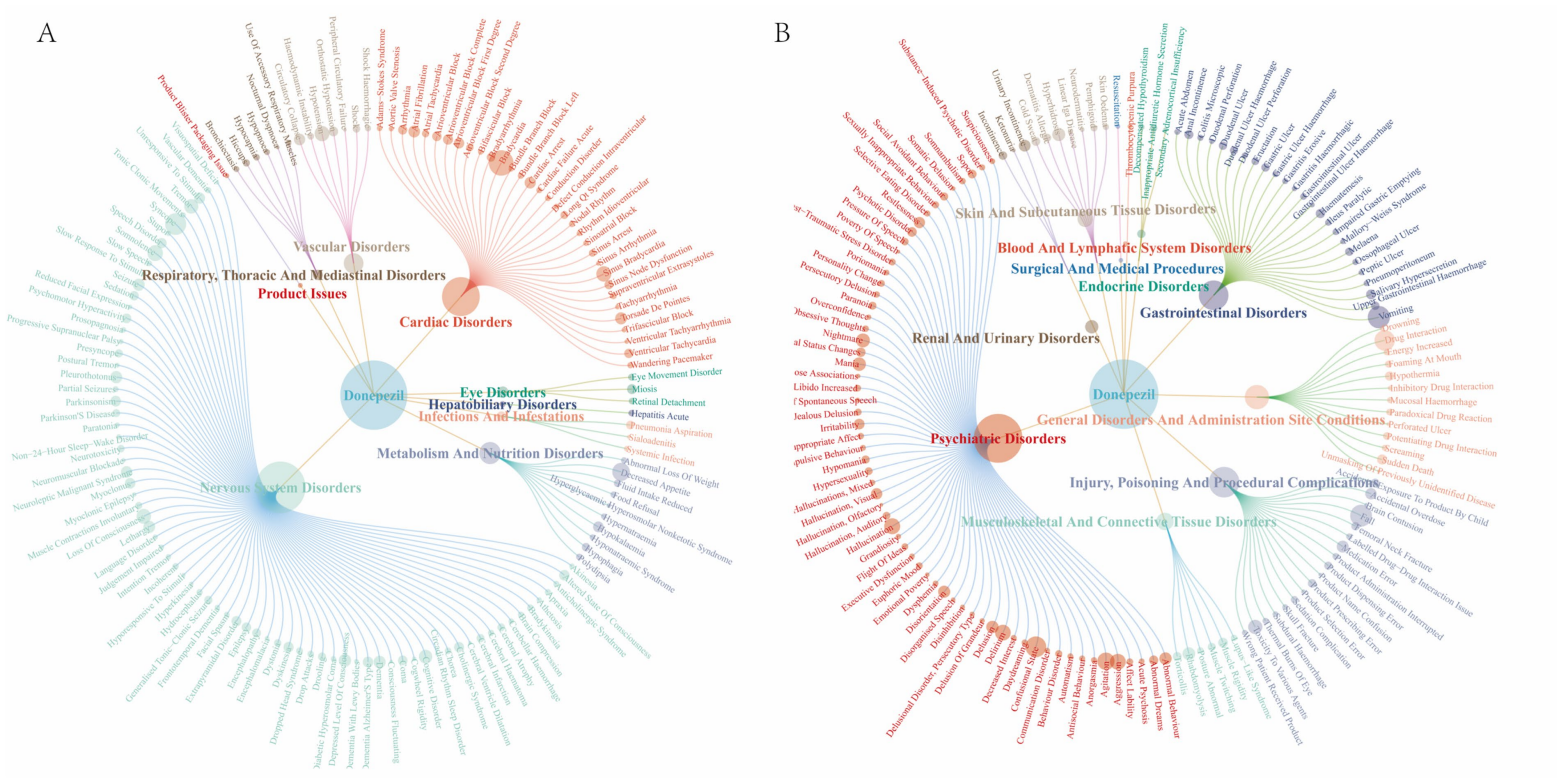
Donepezil SOC and PT distribution network map.

The signal detection of donepezil-associated AEs at the SOC level was carried out as shown in Table 3. The categorical mesh plot of the SOCs and PTs that met all four criteria for donepezil was produced, see Figure 4. The pharmacovigilance profile of donepezil was found to impact 26 organ physiological domains, and statistically, the SOCs that were identified to meet all four criteria simultaneously and to show a significant correlation with donepezil AEs were Nervous system disorders (*n* = 5,059, ROR = 2.58, PRR = 2.27, EBGM = 2.27, IC = 1.18), psychiatric disorders (*n* = 4,106, ROR = 3.08, PRR = 2.75, EBGM = 2.75, IC = 1.46), and cardiac disorders (*n* = 2,356, ROR = 3.63, PRR = 3.39, EBGM = 3.39, IC = 1.76). Nervous system disorders were the most frequently observed SOC, and further, Gastrointestinal disorders (*n* = 2,835) and General disorders and administration site conditions (*n* = 2,826) were found to be common and noteworthy SOC categories.

**Table 3:** Signal intensity of SOC-level donepezil AE in the FAERS database.

System organ class	Case numbers (*n* = 26,120)	ROR (95%two sided CI)	PRR (X^2^)	IC (IC025)	EBGM (EBGM05)
Nervous system disorders	5,059	2.58 (2.5–2.66)*	2.27 (3,940.15)*	1.18 (1.14)*	2.27 (2.21)*
Psychiatric disorders	4,106	3.08 (2.98–3.18)*	2.75 (4,844.74)*	2.75 (2.67)*	1.46 (1.41)*
Gastrointestinal disorders	2,835	1.29 (1.24–1.34)*	1.26 (167.1)	0.33 (0.28)*	1.26 (1.22)
General disorders and administration site conditions	2,826	0.57 (0.55–0.59)	0.62 (818.76)	−0.7 (−0.76)	0.62 (0.6)
Cardiac disorders	2,356	3.63 (3.48–3.79)*	3.39 (4,080.87)*	1.76 (1.7)*	3.39 (3.27)*
Injury, poisoning, and procedural complications	1,908	0.75 (0.72–0.79)	0.77 (147.92)	−0.38 (−0.45)	0.77 (0.74)
Investigations	1,294	0.78 (0.74–0.83)	0.79 (73.58)	−0.33 (−0.41)	0.79 (0.76)
Metabolism and nutrition disorders	909	1.63 (1.53–1.74)*	1.61 (213.87)	0.69 (0.59)*	1.61 (1.52)
Musculoskeletal and connective tissue disorders	798	0.56 (0.52–0.6)	0.57 (265.74)	−0.8 (−0.9)	0.57 (0.54)
Skin and subcutaneous tissue disorders	694	0.47 (0.44–0.51)	0.49 (391.92)	−1.03 (−1.14)	0.49 (0.46)
Vascular disorders	682	1.21 (1.12–1.3)*	1.2 (23.69)	0.27 (0.15)*	1.2 (1.13)
Respiratory, thoracic, and mediastinal disorders	599	0.46 (0.43–0.5)	0.48 (362.08)	−1.07 (−1.19)	0.48 (0.45)
Renal and urinary disorders	436	0.9 (0.82–0.99)	0.9 (4.81)	−0.15 (−0.29)	0.9 (0.83)
Infections and infestations	416	0.29 (0.26–0.32)	0.3 (722.22)	−1.74 (−1.88)	0.3 (0.28)
Eye disorders	217	0.41 (0.35–0.46)	0.41 (187.57)	−1.28 (−1.48)	0.41 (0.37)
Blood and lymphatic system disorders	216	0.48 (0.42–0.54)	0.48 (123.35)	−1.06 (−1.25)	0.48 (0.43)
Hepatobiliary disorders	205	0.85 (0.74–0.98)	0.85 (5.38)	−0.23 (−0.43)	0.85 (0.76)
Social circumstances	122	1.07 (0.89–1.27)	1.07 (0.49)	0.09 (−0.17)	1.07 (0.92)
product issues	84	0.2 (0.16–0.25)	0.2 (269.75)	−2.31 (−2.62)	0.2 (0.17)
Neoplasms benign, malignant, and unspecified (incl cysts and polyps)	78	0.11 (0.09–0.14)	0.11 (562.43)	−3.15 (−3.48)	0.11 (0.09)
Surgical and medical procedures	77	0.21 (0.17–0.27)	0.22 (223.64)	−2.22 (−2.54)	0.22 (0.18)
Ear and labyrinth disorders	72	0.63 (0.5–0.8)	0.64 (15.11)	−0.65 (−0.99)	0.64 (0.52)
Endocrine disorders	60	0.89 (0.69–1.15)	0.89 (0.77)	−0.16 (−0.53)	0.89 (0.72)
Immune system disorders	42	0.14 (0.1–0.19)	0.14 (217.81)	−2.8 (−3.24)	0.14 (0.11)
Reproductive system and breast disorders	24	0.11 (0.07–0.16)	0.11 (171.7)	−3.17 (−3.74)	0.11 (0.08)
Congenital, familial, and genetic disorders	5	0.06 (0.03–0.15)	0.06 (70.75)	−4 (−5.18)	0.06 (0.03)

*AEs, that are not mentioned in the drug label.

**Figure 5:**
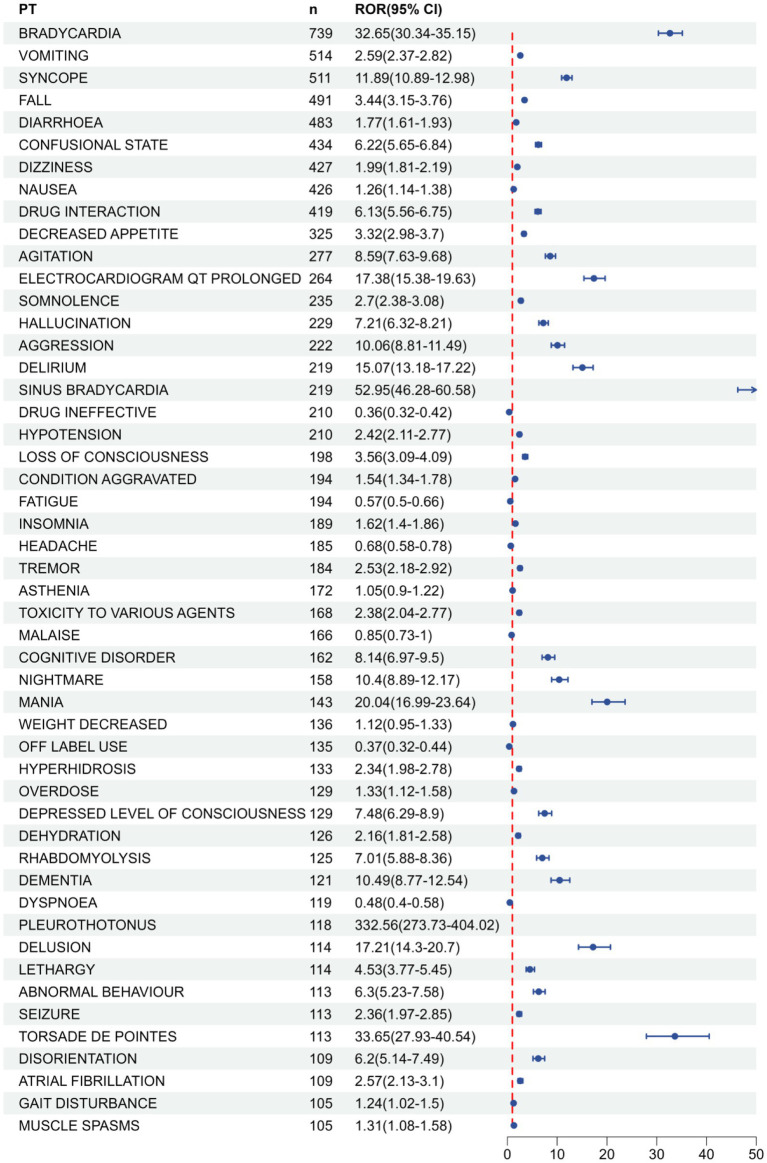
FAERS database top 50 forest plots of AE frequency of donepezil at PT level. Panel **(B)** is a continuation chart of Panel **(A)**.

Ranking the AEs that met all four screening criteria were independently conducted, relying on signal frequency and intensity derived from the most sensitive ROR algorithm’s outcomes, as detailed in Table 4 and Figure 5. Meanwhile, due to the high sensitivity of ROR, we selected the top 10 AEs signals with the strongest ROR signals, as shown in Table 5. In the results, vomiting (*n* = 514), diarrhea (*n* = 483), and nausea (*n* = 426) were identified as the most prevalent AEs, consistent with the product labeling and clinical trial findings. The potential risk signals were demonstrated by AEs such as pleurothotonus (ROR = 332.56, PRR = 331.06, IC = 8.16, EBGM = 285.67), sinus bradycardia (ROR = 52.95, PRR = 52.51, IC = 5.68, EBGM = 51.24), torsade de pointes (ROR = 33.65, PRR = 33.51, IC = 5.04, EBGM = 32.99), bradycardia (ROR = 32.65, PRR = 31.76, IC = 4.97, EBGM = 31.3), mania (ROR = 20.04, PRR = 19.93, IC = 4.3, EBGM = 19.97), and electrocardiogram qt prolonged (ROR = 17.38, PRR = 17.21, IC = 4.09, EBGM = 17.08). Moreover, unanticipated significant AEs were found when compared to the latest FDA drug insert, including falls (ROR = 3.44, PRR = 3.39, IC = 1.76, EBGM = 3.39), delirium (ROR = 15.07, PRR = 14.95, IC = 3.89, EBGM = 14.85), hypotension (ROR = 2.42, PRR = 2.4, IC = 1.26, EBGM = 2.4), loss of consciousness (ROR = 3.56, PRR = 3.54, IC = 1.82, EBGM = 3.53), tremor (ROR = 2.53, PRR = 2.52, IC = 1.33, EBGM = 2.51), cognitive disorder (ROR = 8.14, PRR = 8.1, IC = 3.01, EBGM = 8.07), mania (ROR = 20.04, PRR = 19.93, IC = 4.3, EBGM = 19.97), hyperhidrosis (ROR = 2.34, PRR = 2.34, IC = 1.22, EBGM = 2.34), pleurothotonus (ROR = 332.56, PRR = 331.06, IC = 8.16, EBGM = 285.67), and disorientation (ROR = 6.2, PRR = 6.18, IC = 2.62, EBGM = 6.16). It should be noted that diarrhea, dizziness, nausea, fatigue, insomnia, headache, muscle spasms, and dehydration did not fulfill at least one criterion among the four algorithms in the labeling.

**Table 4:** Top 50 AE frequencies for donepezil at PT levels in the FAERS database, see Annex 1 for all AEs.

SOC	PTs	Case numbers (*n* = 11,233)	ROR (95%two sided CI)	PRR (X^2^)	IC (IC025)	EBGM (EBGM025)
Cardiac disorders	Bradycardia	739	32.65 (30.34–35.15)	31.76 (21,703.29)	31.3 (29.42)	4.97 (4.86)
Gastrointestinal disorders	Vomiting	514	2.59 (2.37–2.82)	2.56 (490.6)	2.56 (2.38)	1.35 (1.23)
Nervous system disorders	Syncope	511	11.89 (10.89–12.98)	11.68 (4,969.11)	11.62 (10.79)	3.54 (3.41)
Injury, poisoning, and procedural complications	Fall^*^	491	3.44 (3.15–3.76)	3.39 (832.38)	3.39 (3.15)	1.76 (1.63)
Gastrointestinal disorders	Diarrhea	483	1.77 (1.61–1.93)	1.75 (157.46)	1.75 (1.62)	0.81 (0.68)
Psychiatric disorders	Confusional state	434	6.22 (5.65–6.84)	6.13 (1,862.6)	6.11 (5.65)	2.61 (2.47)
Nervous system disorders	Dizziness	427	1.99 (1.81–2.19)	1.97 (206.49)	1.97 (1.82)	0.98 (0.84)
Gastrointestinal disorders	Nausea	426	1.26 (1.14–1.38)	1.25 (21.82)	1.25 (1.16)	0.32 (0.18)
General disorders and administration site conditions	Drug interaction	419	6.13 (5.56–6.75)	6.04 (1,763.55)	6.03 (5.56)	2.59 (2.45)
Metabolism and nutrition disorders	Decreased appetite	325	3.32 (2.98–3.7)	3.29 (519.63)	3.29 (3)	1.72 (1.56)
Psychiatric disorders	Agitation	277	8.59 (7.63–9.68)	8.51 (1,831.77)	8.48 (7.68)	3.08 (2.91)
Investigations	Electrocardiogram qt prolonged	264	17.38 (15.38–19.63)	17.21 (4,000.24)	17.08 (15.42)	4.09 (3.92)
Nervous system disorders	Somnolence	235	2.7 (2.38–3.08)	2.69 (249.86)	2.69 (2.41)	1.43 (1.24)
Psychiatric disorders	Hallucination	229	7.21 (6.32–8.21)	7.15 (1,208.9)	7.13 (6.39)	2.83 (2.64)
Psychiatric disorders	Aggression	222	10.06 (8.81–11.49)	9.98 (1,787.71)	9.94 (8.9)	3.31 (3.12)
Psychiatric disorders	Delirium^*^	219	15.07 (13.18–17.22)	14.95 (2,831.37)	14.85 (13.28)	3.89 (3.7)
Cardiac disorders	Sinus bradycardia	219	52.95 (46.28–60.58)	52.51 (10,795.63)	51.24 (45.78)	5.68 (5.48)
General disorders and administration site conditions	Drug ineffective	210	0.36 (0.32–0.42)	0.37 (233.45)	0.37 (0.33)	−1.44 (−1.64)
Vascular disorders	Hypotension^*^	210	2.42 (2.11–2.77)	2.4 (172.69)	2.4 (2.14)	1.26 (1.07)
Nervous system disorders	Loss of consciousness^*^	198	3.56 (3.09–4.09)	3.54 (360.23)	3.53 (3.14)	1.82 (1.61)
General disorders and administration site conditions	Condition aggravated	194	1.54 (1.34–1.78)	1.54 (36.88)	1.54 (1.37)	0.62 (0.42)
General disorders and administration site conditions	Fatigue	194	0.57 (0.5–0.66)	0.58 (60.49)	0.58 (0.51)	−0.79 (−1)
Psychiatric disorders	Insomnia	189	1.62 (1.4–1.86)	1.61 (43.96)	1.61 (1.43)	0.69 (0.48)
Nervous system disorders	Headache	185	0.68 (0.58–0.78)	0.68 (28.6)	0.68 (0.6)	−0.56 (−0.77)
Nervous system disorders	Tremor^*^	184	2.53 (2.18–2.92)	2.52 (168.23)	2.51 (2.23)	1.33 (1.12)
General disorders and administration site conditions	Asthenia	172	1.05 (0.9–1.22)	1.05 (0.41)	1.05 (0.93)	0.07 (−0.15)
Injury, poisoning, and procedural complications	Toxicity to various agents	168	2.38 (2.04–2.77)	2.37 (133.18)	2.37 (2.09)	1.24 (1.02)
General disorders and administration site conditions	Malaise	166	0.85 (0.73–1)	0.86 (4.08)	0.86 (0.75)	−0.23 (−0.45)
Nervous system disorders	Cognitive disorder^*^	162	8.14 (6.97–9.5)	8.1 (1,004.3)	8.07 (7.09)	3.01 (2.79)
Psychiatric disorders	Nightmare	158	10.4 (8.89–12.17)	10.35 (1,328.15)	10.3 (9.03)	3.36 (3.13)
Psychiatric disorders	Mania^*^	143	20.04 (16.99–23.64)	19.93 (2,547.88)	19.75 (17.2)	4.3 (4.06)
Investigations	Weight decreased	136	1.12 (0.95–1.33)	1.12 (1.74)	1.12 (0.97)	0.16 (−0.08)
Injury, poisoning, and procedural complications	Off label use	135	0.37 (0.32–0.44)	0.38 (141.01)	0.38 (0.33)	−1.41 (−1.66)
Skin and subcutaneous tissue disorders	Hyperhidrosis^*^	133	2.34 (1.98–2.78)	2.34 (101.95)	2.34 (2.03)	1.22 (0.97)
Injury, poisoning, and procedural complications	Overdose	129	1.33 (1.12–1.58)	1.33 (10.57)	1.33 (1.15)	0.41 (0.16)
Nervous system disorders	Depressed level of consciousness	129	7.48 (6.29–8.9)	7.45 (718.53)	7.43 (6.43)	2.89 (2.64)
Metabolism and nutrition disorders	Dehydration	126	2.16 (1.81–2.58)	2.16 (78.22)	2.16 (1.86)	1.11 (0.85)
Musculoskeletal and connective tissue disorders	Rhabdomyolysis	125	7.01 (5.88–8.36)	6.98 (638.52)	6.96 (6.01)	2.8 (2.54)
Nervous system disorders	Dementia	121	10.49 (8.77–12.54)	10.44 (1,028.66)	10.4 (8.95)	3.38 (3.12)
Respiratory, thoracic, and mediastinal disorders	Dyspnoea^*^	119	0.48 (0.4–0.58)	0.48 (66.2)	0.48 (0.42)	−1.05 (−1.31)
Nervous system disorders	Pleurothotonus^*^	118	332.56 (273.73–404.02)	331.06 (33,489.62)	285.67 (242.73)	8.16 (7.88)
Psychiatric disorders	Delusion^*^	114	17.21 (14.3–20.7)	17.13 (1,718.25)	17 (14.57)	4.09 (3.82)
Nervous system disorders	Lethargy^*^	114	4.53 (3.77–5.45)	4.52 (311.72)	4.51 (3.86)	2.17 (1.9)
Psychiatric disorders	Abnormal behavior	113	6.3 (5.23–7.58)	6.27 (499.79)	6.26 (5.36)	2.65 (2.37)
Nervous system disorders	Seizure	113	2.36 (1.97–2.85)	2.36 (88.53)	2.36 (2.02)	1.24 (0.97)
Cardiac disorders	Torsade de pointes	113	33.65 (27.93–40.54)	33.51 (3,507.84)	32.99 (28.23)	5.04 (4.77)
Psychiatric disorders	Disorientation^*^	109	6.2 (5.14–7.49)	6.18 (472.04)	6.16 (5.26)	2.62 (2.35)
Cardiac disorders	Atrial fibrillation	109	2.57 (2.13–3.1)	2.56 (103.56)	2.56 (2.18)	1.35 (1.08)
General disorders and administration site conditions	Gait disturbance^*^	105	1.24 (1.02–1.5)	1.24 (4.85)	1.24 (1.06)	0.31 (0.03)
Musculoskeletal and connective tissue disorders	Muscle spasms	105	1.31 (1.08–1.58)	1.31 (7.56)	1.31 (1.11)	0.39 (0.1)

* AEs, that are not mentioned in the drug label.

**Table 5:** The top 10 AE signals with the strongest ROR signals for donepezil.

SOC	PTs	Case numbers (*n* = 11,233)	ROR (95%two sided CI)
Cardiac disorders	Bradycardia	739	32.65 (30.34–35.15)
Nervous system disorders	Syncope	511	11.89 (10.89–12.98)
Investigations	Electrocardiogram qt prolonged	264	17.38 (15.38–19.63)
Psychiatric disorders	Delirium*	219	15.07 (13.18–17.22)
Cardiac disorders	Sinus bradycardia	219	52.95 (46.28–60.58)
Psychiatric disorders	Mania*	143	20.04 (16.99–23.64)
Nervous system disorders	Dementia	121	10.49 (8.77–12.54)
Nervous system disorders	Pleurothotonus*	118	332.56 (273.73–404.02)
Psychiatric disorders	Delusion*	114	17.21 (14.3–20.7)
Cardiac disorders	Torsade de pointes	113	33.65 (27.93–40.54)

*AEs, that are not mentioned in the drug label.

### 3.3 Time-to-onset analysis

**Figure 6:**
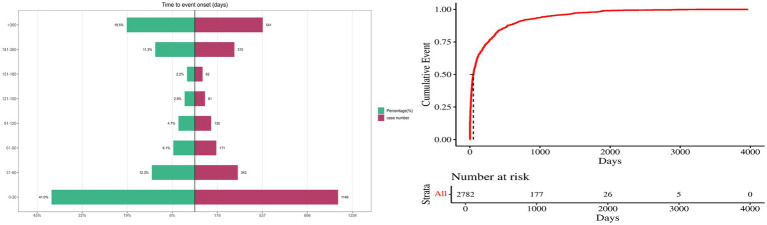
Time to onset of donepezil-related AEs.

Seizure times associated with donepezil-related events were collected, while cases with unreported, misreported, or missing seizure times were excluded from the analysis. A total of 2,782 ICSRs fulfilled the inclusion criteria. The average time to onset was 232 days; the median seizure time was 49 days, with an interquartile range of 11–265.75 days. It was observed that 41.0% (*n* = 1,140) of ICSRs occurred within the first month of donepezil use. Importantly, 19.4% of all cases (*n* = 541) involved AEs occurring more than 1 year after therapy began, as shown in Figure 6.

### 3.4 Gender signaling differences in donepezil

**Figure 7:**
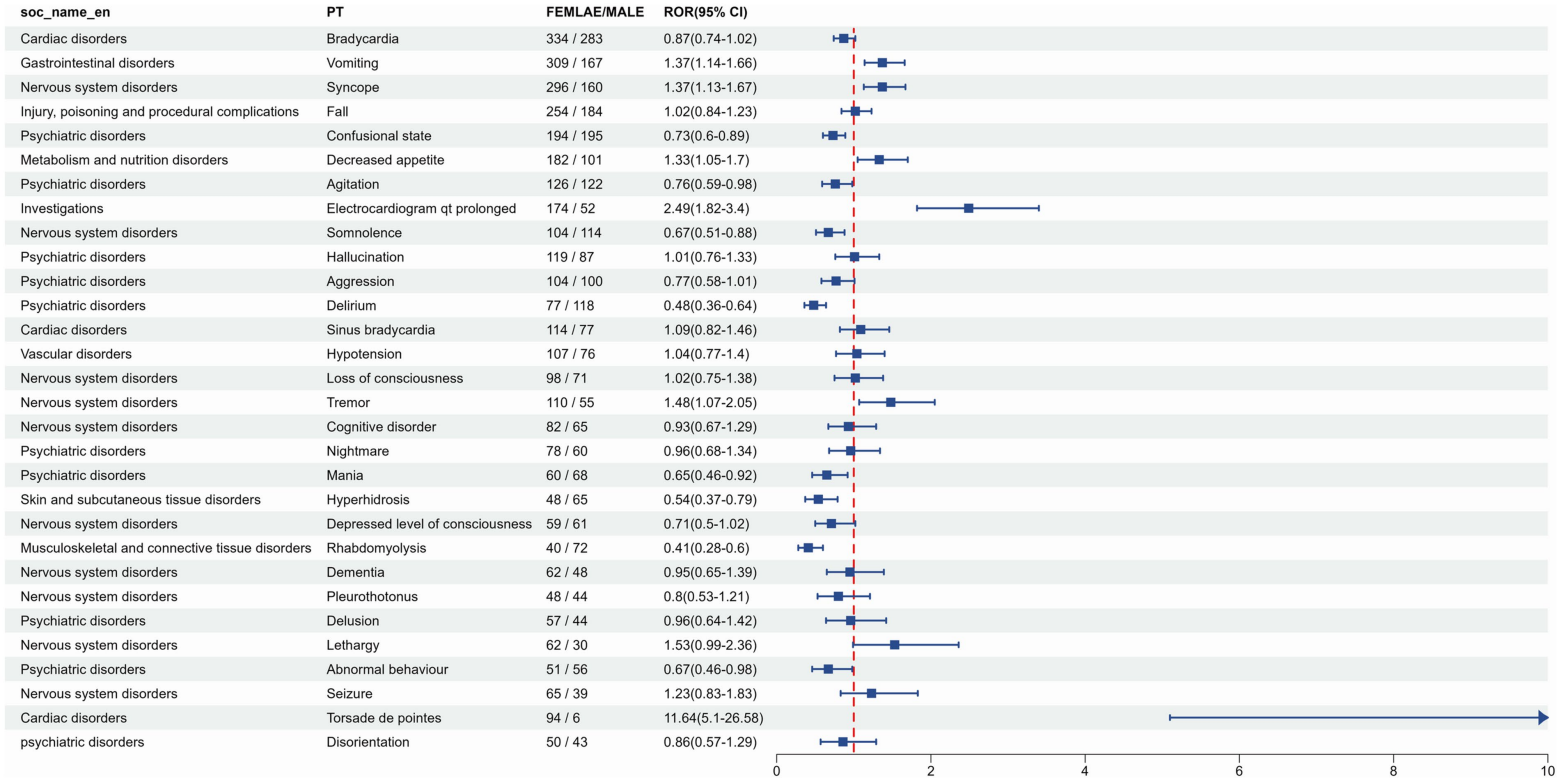
Forest map for gender subgroup analysis, the full PT signals for males and females are shown in Annex 2, 3, respectively, and all date in Annex 4.

Based on the reported gender data of donepezil, 4,591 (51.3%) females were compared to 3,187 (35.6%) males. To determine whether gender impacts the adverse reactions of donepezil, the ROR algorithm was used, and PTs meeting all four algorithmic criteria and ranking within the top 30 in frequency were identified. These PTs exhibited varying incidence rates between males and females. Categorization by SOC was presented in Figure 7, and AEs such as vomiting, syncope, appetite loss, electrocardiogram QT prolongation, tremor, drowsiness, seizures, and tip-torsade de pointes were observed more frequently in females, with tip-torsade de pointes demonstrating particular gender specificity. However, high-risk AEs like altered states of consciousness, agitation, somnolence, aggression, delirium, mania, hyperhidrosis, decreased consciousness, rhabdomyolysis, and abnormal behavior were more prevalent among males.

## 4 Discussion

Alzheimer’s disease is recognized as one of the leading health issues in a globally aging population, making the safety evaluation of its treatment agents essential. Donepezil, considered a primary AChEI, is known to improve cognitive function, although concerns remain about its long-term safety. Pharmacovigilance related to adverse events, such as donepezil-induced bradycardia and its combination with memantine in adult populations, has been addressed in several studies in recent years (10, 20). Despite this, a comprehensive safety assessment of donepezil-induced AE has not been presented. In this study, post-marketing AE data of donepezil were collected and evaluated using a real-world pharmacovigilance data system. This study aimed to detect novel and substantial risk signals and improve the safety of clinical pharmacotherapy.

This study included 8,943 ICSRs associated with donepezil and 26,120 AE reports. The results revealed that donepezil-associated AEs involved 26 system organ classifications (SOCs), with Nervous system disorders (5,059 cases, ROR = 2.58), psychiatric disorders (4,106 cases, ROR = 3.08), and cardiac disorders (2,356 cases, ROR = 3.63) as the main affected systems. At the preferred term (PT) level, the most frequently reported AEs included bradycardia (*n* = 739, ROR = 32.65), vomiting (*n* = 514, ROR = 2.59), and syncope (*n* = 511, ROR = 11.89). Gastrointestinal events such as diarrhea (*n* = 483, ROR = 1.77) and nausea (*n* = 426, ROR = 1.26) were consistent with drug inserts and clinical trial findings (21). However, this study also identified novel AEs such as falls (ROR = 3.44), delirium (ROR = 15.07), pleurothotonus (ROR = 332.56), disorientation (ROR = 6.2), and hypotension (ROR = 2.42) through data mining. These findings support existing clinical understanding and extend awareness of donepezil’s potential risks.

The results of this study closely match previous clinical trials and observational studies. For instance, bradycardia (ROR = 32.65) emerged as the most frequent AE associated with donepezil treatment (22), sinus bradycardia (ROR = 52.95) presented a significantly higher risk value than other cardiac events, indicating a specific influence of cholinesterase inhibition on sinus node function. Donepezil’s central mechanism of action involves hyperactivation of the cholinergic system, which increases parasympathetic activity *via* inhibition of AChE, potentially resulting in sinus node suppression and delayed atrioventricular conduction (23). Recent animal studies have demonstrated that donepezil extends cardiomyocyte action potential duration and shows a strong association with electrocardiogram qt prolonged (ROR = 17.38) and tip-torsade de pointes (ROR = 33.65) (24). Pleurothotonus (ROR = 332.56), a highly rare event identified in this study, remains etiologically unclear. Suggested mechanisms involve an imbalance in dopaminergic-cholinergic systems and roles for noradrenergic and serotonergic neurotransmission (25). The primary mechanism is thought to involve dopaminergic-cholinergic dysregulation within the basal ganglia, a pathway critically involved in motor control. Acetylcholinesterase inhibitors like donepezil enhance central cholinergic tone, which may disrupt the delicate balance between dopaminergic and cholinergic systems, leading to dystonic manifestations such as pleurothotonus (26). Additionally, emerging evidence suggests the involvement of noradrenergic and serotonergic pathways. Noradrenergic dysfunction, particularly originating from the locus coeruleus, may contribute to impaired postural control, while serotonergic system modulation can influence complex motor outputs via interactions with basal ganglia circuitry. This multi-transmitter hypothesis provides a plausible framework for pleurothotonus, positioning it as a rare but severe adverse effect stemming from disrupted neuromodulatory integration (27). Furthermore, the high signal intensity of cognitive disorder (ROR = 8.14) versus mania (ROR = 20.04) challenged conventional knowledge, suggesting that donepezil may modulate psychiatric behavior *via* intricate neurotransmitter interactions.

This study identified significantly higher donepezil-associated AEs in women (51.3%) and elderly patients over 65 years old (67.7%), consistent with the known epidemiologic profile (28). Pathophysiologic changes associated with endocrine transitions, such as menopause, which are both age- and sex-related, may contribute to the higher prevalence of AD in female patients (29). Female patients also reported more adverse effects of donepezil than male patients, potentially due to pharmacokinetic differences; lower activity of cytochrome P450 enzymes such as CYP2D6 in females reduces clearance and elevates blood concentrations of donepezil (30). Moreover, factors such as reduced hepatic and renal function and the use of multiple medications (e.g., beta-blockers, anticholinergics) in the elderly may further increase AE risk (31). In this study, sinus bradycardia (ROR = 52.95) accelerated dementia progression when co-administered with bladder anticholinergic drugs, which was consistent with previous findings (32). Most reports were submitted from developed countries. Although a decline in dementia prevalence has been suggested in high-income countries, the evidence remains inconclusive (33). Furthermore, data from the USA and GBR, which accounted for more than half of the total reports, were filtered for trend comparison. To simplify comparison, the whole dataset was reduced by one-quarter. While the overall trend in AE reporting has increased in recent years, a decrease in reports from the USA and GBR was observed, consistent with the hypothesis of declining incidence in high-income nations.

In the sex subgroup analysis, a higher likelihood of torsade de pointes (ROR = 33.65) and electrocardiogram qt prolonged (ROR = 17.38) was observed in women, while men were more often reported to experience agitation (ROR = 10.06) and delirium (ROR = 15.07). These findings may be attributed to the influence of sex hormones on cardiac ion channels, such as hERG potassium channels, and neurotransmitter pathways, such as the dopaminergic system (34). Furthermore, it has been reported that estrogens (35) are thought to prolong myocardial repolarization by inhibiting hERG potassium channels, whereas androgens may stimulate dopaminergic signaling, potentially accounting for the sex-specific AE patterns, including a greater incidence of cardiovascular effects in females than males (34, 36). Therefore, clinical decision-making must consider gender to reduce the likelihood of adverse outcomes. Furthermore, social factors such as women’s more frequent visits to the doctor and men’s lesser attention to their health problems may also influence the reporting bias, which needs to be verified in a prospective study (37, 38).

Among the unintended AEs, falls (ROR = 3.44) and pleurothotonus (ROR = 332.56) were particularly concerning. Falls represented the most frequently reported unintended AE in the PT dataset, consistent with the findings of other studies (39, 40). Although no mechanistic evidence is currently available, an association with cerebral ischemia from cardiac-related AEs is hypothesized, with elderly comorbidities like muscle atrophy and unstable gait increasing risk (41, 42). Pleurothotonus, an abnormal body posture defined by a backward tilt with the head and feet bent posteriorly, has a significant risk value (ROR = 332.56) and a substantial sample size (118 cases) in this study, with pertinent instances reported (43). The mechanism remains uncertain and requires validation through further studies. Moreover, there were unintended AE signals including disorientation (ROR = 6.2), cognitive function (ROR = 8.14), delirium (ROR = 15.07), hypotension (ROR = 2.42), loss of consciousness (ROR = 3.56), and tremor (ROR = 2.53), which require validation in future studies.

This study identified a significant difference between the incidence of AEs and the administration of donepezil, with a median onset time of AEs at 49 days and temporal distribution characteristics indicating that 41% occurred within the first month. This suggests a need for improved clinical monitoring during the initial treatment phase, particularly within the first month, focusing on cardiovascular and neuropsychiatric assessments of the primary affected systems of the SOC. we recommend enhanced clinical monitoring during this period, including baseline and follow-up electrocardiograms, assessment of orthostatic vital signs, and evaluation of neuropsychiatric symptoms. However, 19.4% of ICSR incidents were delayed, occurring after 1 year of donepezil treatment. These results indicate the need for monitoring for AEs even after 1 year of treatment, emphasizing the significance of long-term follow-up. We advised that, particularly in elderly patients and those on polypharmacy, to mitigate risks such as falls (44), cognitive decline, and cardiac conduction abnormalities.

## 5 Limitations

This study has several limitations. The reporting of AEs occurred spontaneously, which led to potential underreporting or overreporting within the FAERS database and substantial missing data. Reporters included health professionals such as physicians (*n* = 594, 17.1%) and pharmacists (*n* = 1,100, 31.7%), as well as non-health professionals such as consumers. Although most data originated from professionals, reporting can still be biased. Moreover, the FAERS database may present systemic bias (45). Furthermore, the overrepresentation of reports from high-income countries (e.g., USA and UK) may reflect disparities in healthcare access, pharmacovigilance infrastructure, and reporting behavior rather than true differences in AE incidence. Similarly, the higher proportion of female reporters may be influenced by gender-specific health-seeking behaviors. These factors underscore the need for cautious interpretation of the data and highlight the importance of enhancing global pharmacovigilance coverage. The limited sample size also could lead to the omission of some rare adverse reactions. Therefore, further empirical research and additional reported data are required to validate these findings. However, the current study provides meaningful insights to inform clinicians and patients about the AEs associated with donepezil and assist in selecting more optimal therapeutic agents.

## 6 Conclusion

In conclusion, this study systematically revealed the safety profile of donepezil in the real world, confirming that its common AEs were consistent with the drug labeling. It also identified unintended risk signals such as falls, pleurothotonus, and disorientation. Gender and age differences in adverse effects were also identified. Future research should prioritize prospective or case–control studies to validate signals of serious or unexpected AEs identified in this analysis, such as pleurothotonus, QT prolongation, and gender-specific neuropsychiatric events. Such studies would help establish causality and inform risk mitigation strategies. In clinical practice, individualized monitoring should be implemented for female, elderly, and co-morbid patients, and attention should be given to potential drug interaction effects. This study provides real-world evidence that may support updates to the donepezil prescribing information. Our study identified several unintended but significant AEs—such as falls, pleurothotonus, delirium, and disorientation—that are not currently highlighted in the official FDA prescribing information for donepezil. These findings underscore the need to update the drug label to include these potential risks, which could aid clinicians in early recognition and management. Furthermore, the strong signals for cardiac events like sinus bradycardia and QT prolongation, particularly in women and the elderly, suggest that pre-treatment cardiac assessment and periodic monitoring should be emphasized in clinical guidelines. By integrating these real-world safety signals into drug labeling and treatment protocols, healthcare providers can better tailor therapy, mitigate risks, and improve patient outcomes in the management of Alzheimer’s disease.

## Data Availability

The original contributions presented in the study are included in the article/Supplementary material, further inquiries can be directed to the corresponding author.
